# Case Report: Significant Response to the Combination of Lenvatinib and Immune Checkpoint Inhibitor in a Patient With Heavily Pretreated Metastatic Triple Negative Breast Cancer

**DOI:** 10.3389/fonc.2020.582185

**Published:** 2021-01-11

**Authors:** Jin Sun Lee, Susan E. Yost, Yuan Yuan

**Affiliations:** Department of Medical Oncology & Molecular Therapeutics, City of Hope Comprehensive Cancer Center and Beckman Research Institute, Duarte, CA, United States

**Keywords:** triple negative breast cancer, lenvatinib, immune checkpoint inhibitors, pembrolizumab, personalized treatment

## Abstract

**Background:**

Triple negative breast cancer (TNBC) has poor prognosis without targetable mutations. The combination of lenvatinib and pembrolizumab has shown clinical activity in different types of solid tumors.

**Case Presentation:**

We report a case of one patient with metastatic TNBC who has been heavily pretreated. The patient had been treated with multiple lines (≥ 8 lines) of chemotherapy without durable clinical responses. Her tumor regressed significantly under the combination of lenvatinib and immune checkpoint inhibitor, and remains stable for 10 months.

**Conclusions:**

The combination of lenvatinib and immune checkpoint inhibitor may have significant clinical activity in selective patients with heavily pretreated metastatic TNBC.

## Introduction

Triple negative breast cancer (TNBC) is associated with impaired clinical outcome compared with other types of breast cancer (1). While breast cancer has improved survival outcomes contributed by hormone and HER2 targeted therapy in the past decades, TNBC remains a non-targetable disease without significant advances in survival outcomes (2). The median overall survival (OS) of metastatic TNBC (mTNBC) is about 12 months with treatment (3).

Currently, mTNBC has limited treatment options including cytotoxic chemotherapy, and the response is poor after prior treatment. A recent clinical trial of the PD-L1 inhibitor atezolizumab combined with nab-paclitaxel demonstrated a significant clinical benefit, with median duration of response of 8.5 months and median progression free survival (PFS) prolonged by 2.5 months in PD-L1 positive group (4). The results are not fulfilling considering this is a first line therapy.

Lenvatinib is a multiple kinase inhibitor against VEGFR1, VEGFR2, and VEGFR3 that has been used as a single agent for advanced thyroid cancer and hepatocellular carcinoma (HCC). Its single use has not been proven for breast cancer. Immune checkpoint inhibitors (ICIs) are extensively used in different types of malignant tumors, and the PD-1 inhibitor pembrolizumab has been studied as a single agent showing a modest response in heavily pretreated mTNBC (5, 6). The early clinical trial of lenvatinib combined with pembrolizumab has demonstrated antitumor activity in multiple solid tumors including advanced endometrial cancer (7, 8). A phase 2 study of lenvatinib plus pembrolizumab in previously treated solid tumors including mTNBC is ongoing (NCT03797326), and the prelim result was recently presented (9).

Herein, we report a case of the patient with heavily pretreated mTNBC who achieved remarkable responses to the combination of lenvatinib and immune checkpoint inhibitors. The patient provided consent to publish their information and images.

## Case

The patient is a 47 years old female with a history of childhood acute lymphoblastic leukemia at age 15, who was in remission after intensive chemotherapy followed by allogenic hematopoietic stem cell transplantation. Patient was diagnosed with breast cancer in July 2013. The initial stage was cT3N2 and pathology was reported as TNBC. She was treated with neoadjuvant chemotherapy (docetaxel and cyclophosphamide) followed by skin sparing mastectomy in October 2013. She received adjuvant chemotherapy (epirubicin and vinorelbine) until March 2014.

Due to rising CA 27-29 in September 2015, PET/CT was performed and showed new lymphadenopathy in the left supra- and infraclavicular and subpectoral area, as well as left axilla. Pathology from fine needle aspiration confirmed mTNBC. She was started on the first line capecitabine in December 2015. She received “immunotherapy (agent name was unknown)” in Germany in 2016 along with intermittent capecitabine. Due to progression of the disease she was enrolled in a clinical trial (unknown name) in July 2016, but developed progression in September 2016. Since then she was treated with multiple lines including third line of carboplatin and gemcitabine started in October 2016, fourth line of pembrolizumab in July 2017, fifth line of carboplatin and nab-paclitaxel in October 2017, sixth line of eribulin in August 2018, seventh line of bicalutamide for AR positivity of the tumor in January 2019, ixabepilone in February 2019, paclitaxel with palliative radiation in April 2019, and nab-paclitaxel and atezolizumab in July 2019.

**Figure 1:**
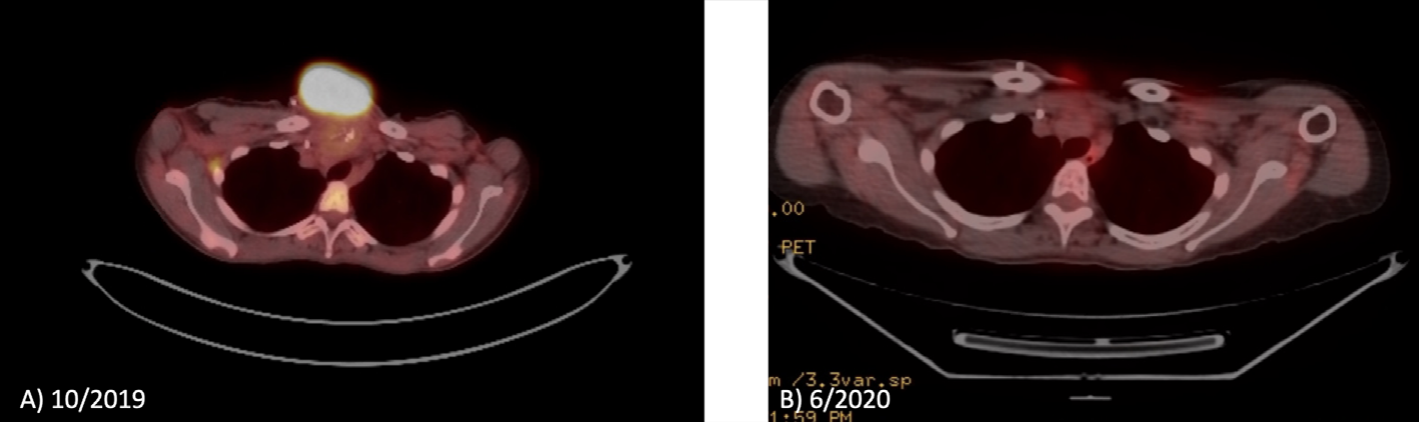
PET/CT before and after lenvatinib plus pembrolizumab on patient 1. **(A)** PET/CT demonstrates the chest well tumor before treatment. **(B)** PET/CT demonstrates near complete response of the chest well tumor.

**Figure 2:**
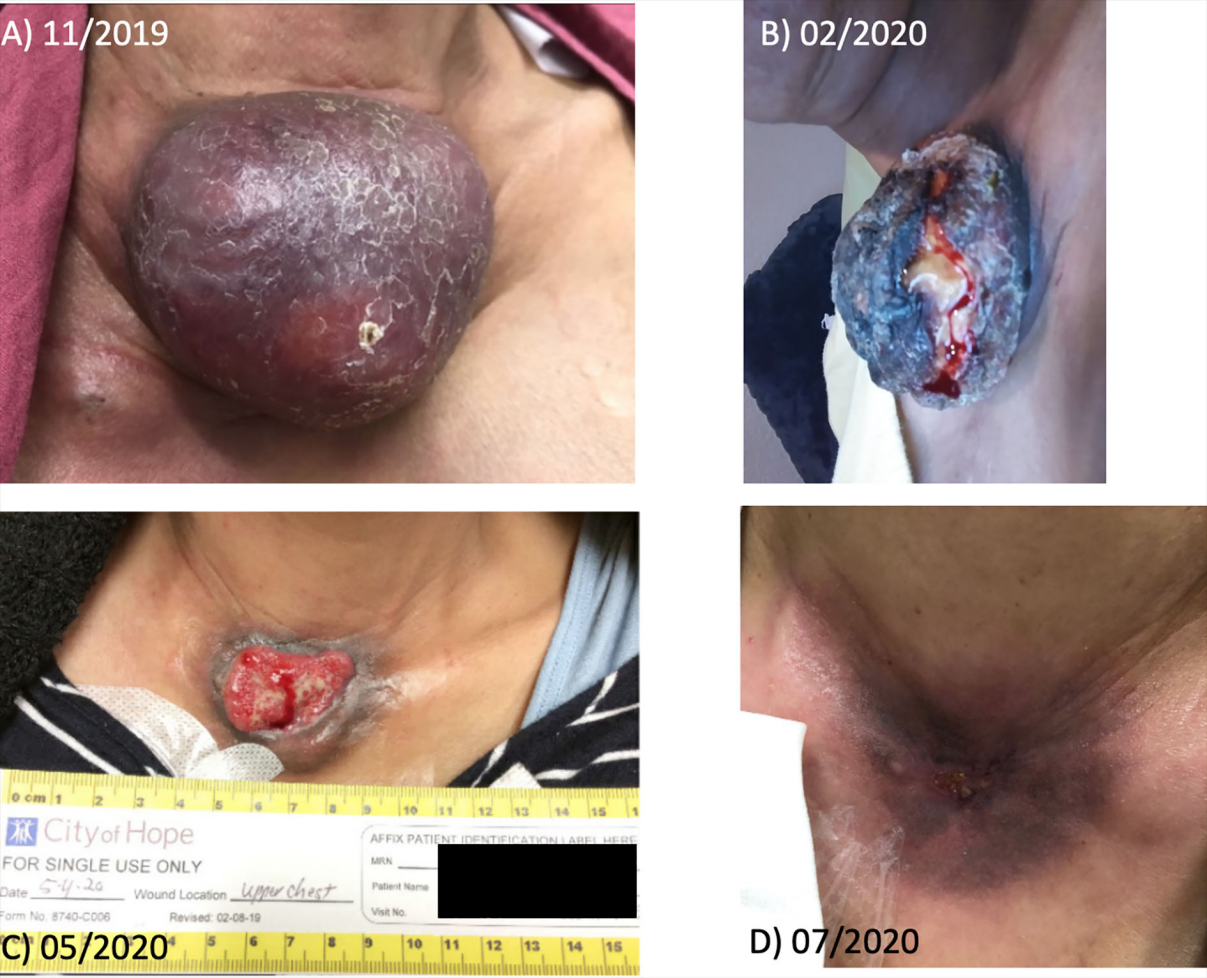
Gross tumor before and after treatment with lenvatinib plus pembrolizumab of patient 1. **(A)** shows the initial tumor. **(B–D)** shows the gross change of the tumor by the timeline.

PET/CT in October 2019 showed increased anterior chest wall mass, right axillary lymph nodes, and mesenteric lymph nodes. Patient visited our institution to seek second opinion in November 2019, and lenvatinib combined with an ICI was recommended. Consequently, the patient was started with lenvatinib 20mg p.o. daily along with the continuation of atezolizumab 840 mg i.v. every 2 weeks from January 2020. After 4 weeks of lenvatinib use, the dose of lenvatinib reduced by 50% (10 mg daily) due to fatigue per the outside oncology note. Patient transferred her care to our institution in May 2020, then atezolizumab was switched to pembrolizumab. The dose of lenvatinib was increased up to 18 mg daily combined with pembrolizumab without significant fatigue or other toxicities. The patient has experienced grade 2 leukopenia and neutropenia once, which improved to grade 1 without medical intervention. Currently patient is on lenvatinib 18 mg daily plus pembrolizumab with significant response for 10 months. PET/CT and images of gross tumor before treatment with lenvatinib combined with ICI are shown in **Figures 1** and **2**. The pain from a large mass was resolved and the sternal wound was healed, and minimal bleeding from the necrotic tumor was as noted as in **Figure 1**; D is the most recent picture with well healed wound).

## Discussion

The multiple kinase inhibitor lenvatinib has anti-angiogenic activity that inhibits vascular endothelial growth factor receptors (VEGFR) 1-3, fibroblast growth factor receptors (FGFR) 1-4, platelet-derived growth factor receptor-a (PDGFRa), tyrosine-kinase receptor KIT, and rearranged during transfectin receptor (RET). Lenvatinib has shown activity in different types of tumors with manageable toxicity profiles (10). Lenvatinib was approved for first line treatment in unresectable HCC after its efficacy was proven in a phase 3 trial (11).

In addition to lenvatinib’s anti-angiogenic activity, several studies in mouse tumor models have shown antitumor immune activation and increased antitumor activity when combined with a PD-1 inhibitor. Zhang et al. demonstrated lenvatinib-induced antitumor immunity by enhancing tumor-infiltrating natural killer (NK) cells (12). Kato et al. reported that lenvatinib modulated cancer immunity in the tumor microenvironment by reducing tumor associated macrophages (TAMs) and showed enhanced antitumor activity by activation of the interferon pathway when combined with a PD-1 inhibitor (13).

The efficacy of lenvatinib combined with pembrolizumab has been evaluated in multiple types of solid tumors (8). A study that included metastatic renal cell carcinoma, endometrial cancer, squamous cell carcinoma of the head and neck, melanoma, non-small cell lung cancer, or urothelial cancer that progressed after approved therapies (or did not have available standard treatment), demonstrated promising antitumor activity with manageable toxicity. The combination of lenvatinib and pembrolizumab has been approved by the FDA for endometrial cancer based on KEYNOTE-146 trial. A total of 108 patients who had previously treated for advanced endometrial cancer were enrolled in this study. The objective response rate (ORR) at 24 weeks was 38.0%, and median PFS was 7.4 months (14).

ICIs are an emerging treatment option for solid tumors, and the efficacy of ICIs are currently being evaluated for mTNBC. The KEYNOTE-086 trial evaluated pembrolizumab single agent for mTNBC and demonstrated an ORR of 21.4% in a previously untreated group and 5.5% in a heavily pretreated group (6, 15). A phase 1 trial with the single agent atezolizumab reported similar results. In this study, the ORRs were 24% in first-line but only 6% in second or greater lines (16). The poor response rates from single agent ICIs, especially in pretreated mTNBC, have led to ICI-based combination treatments (**Table 1**).

**Table 1:** Clinical trials evaluating ICIs combined treatment for triple negative breast cancer.

Trial	Line of Treatment	Medications	Number of patients	Results
IMPassion 130 (4)	1^st^ line	Nab-paclitaxel +/- atezolizumab	450 vs 449	mPFS of 7.2 and 5.5 months
ENHANCE-1 (17)	≤2 prior lines	Eribulin + pembrolizumab	149	ORR of 23.4% (25.8% with 0 prior line and 21.8% with 1–2 prior lines group)
KEYNOTE-355 (18)	1^st^ line	Chemotherapy +/-Pembrolizumab	566 vs 281	mPFS of 7.5 vs 5.6 months
TONIC (19)	Previously treated	Induction with low-dose chemotherapy, irradiationor no induction followed by nivolumab	70	ORR of 17% with no induction, 8% with radiation, 8% with cyclophosphamide, 23% with cisplatin, 35% with doxorubicin
IMPassion 131 (20)	1^st^ line	Paclitaxel +/- atezolizumab	431 vs 220	mPFS 5.7 vs 5.6 months
LEAP-005 (9)	2^nd^ and 3^rd^ line	Lenvatinib + pembrolizumab	31 of TNBC*	ORR of 29%

mPFS, median progression free survival; ORR, objective response rate; *Multicohort study with 31 patients with TNBC.

Recent studies have emphasized the critical role of the tumor microenvironment (TME) (21). The presence of CD8+ tumor-infiltrating lymphocytes that are reactive to clonal neoantigens are associated with durable clinical benefit of the treatment with ICIs (22). In contrast, TAMs have an immunosuppressive function which can be a potential therapeutic target (23, 24). Activation of NK cells has also been associated with prolonged survival and increased response to ICIs (25). The immunomodulatory property of lenvatinib may sensitize TME to ICIs and improve efficacy when combined with ICIs (12, 13).

The patient in this case showed significant responses to lenvatinib combined with ICIs, after treated with 8 lines of chemotherapy without any significant response. The combination of lenvatinib and ICI requires further investigation to identify predictive biomarkers for response and to provide personalized treatment for patients with metastatic TNBC.

## Data Availability Statement

The original contributions presented in the study are included in the article/supplementary material. Further inquiries can be directed to the corresponding author.

## Ethics Statement

Written informed consent was obtained from the individual(s) for the publication of any potentially identifiable images or data included in this article.

## Author Contributions

All authors contributed to the article and approved the submitted version. JL designed the report. JL and SY wrote the manuscript. JL and SY collected the patient’s clinical data. YY were responsible for the conception and revision of the manuscript. YY carried out the clinical management of the patient.

## Conflict of Interest

YY has contracted research sponsored by Merck, Eisai, Novartis, Puma, Genentech, and Pfizer; is a consultant for Puma, and is on the Speakers Bureau for Eisai.

The remaining authors declare that the research was conducted in the absence of any commercial or financial relationships that could be construed as a potential conflict of interest.
